# The Role of the Autonomic Nervous System in the Pathophysiology of Obesity

**DOI:** 10.3389/fphys.2017.00665

**Published:** 2017-09-14

**Authors:** Daniela Guarino, Monica Nannipieri, Giorgio Iervasi, Stefano Taddei, Rosa Maria Bruno

**Affiliations:** ^1^Department of Clinical and Experimental Medicine, University of Pisa Pisa, Italy; ^2^Institute of Clinical Physiology of CNR Pisa, Italy; ^3^Scuola Superiore Sant'Anna Pisa, Italy

**Keywords:** autonomic nervous system, obesity, gut hormones, adipose tissue, energy expenditure, weight loss, vagal nerve stimulation, vagal nerve blockade

## Abstract

Obesity is reaching epidemic proportions globally and represents a major cause of comorbidities, mostly related to cardiovascular disease. The autonomic nervous system (ANS) dysfunction has a two-way relationship with obesity. Indeed, alterations of the ANS might be involved in the pathogenesis of obesity, acting on different pathways. On the other hand, the excess weight induces ANS dysfunction, which may be involved in the haemodynamic and metabolic alterations that increase the cardiovascular risk of obese individuals, i.e., hypertension, insulin resistance and dyslipidemia. This article will review current evidence about the role of the ANS in short-term and long-term regulation of energy homeostasis. Furthermore, an increased sympathetic activity has been demonstrated in obese patients, particularly in the muscle vasculature and in the kidneys, possibily contributing to increased cardiovascular risk. Selective leptin resistance, obstructive sleep apnea syndrome, hyperinsulinemia and low ghrelin levels are possible mechanisms underlying sympathetic activation in obesity. Weight loss is able to reverse metabolic and autonomic alterations associated with obesity. Given the crucial role of autonomic dysfunction in the pathophysiology of obesity and its cardiovascular complications, vagal nerve modulation and sympathetic inhibition may serve as therapeutic targets in this condition.

## Introduction

Obesity is a challenge for global public health. The worldwide prevalence of obesity has nearly doubled in the past decades (World Health Organization). Obesity may induce the onset of other conditions leading to overt cardiovascular disease, such as glucose intolerance, dyslipidemia, impaired glucose tolerance and type 2 diabetes, hypertension, and kidney failure (Martin-Rodriguez et al., 2015; Soares et al., 2015).

In this framework, there is a strong need to reach a deeper understanding of the basic mechanisms coupling energy balance with glucose homeostasis (Flier, 2001; Obici and Rossetti, 2003), in order to develop new treatments able to counteract obesity and thus decrease the risk of cardiovascular disease. The autonomic nervous system (ANS) plays a major role in the integrated regulation of food intake, involving satiety signals and energy expenditure: thus ANS dysregulation might favor body weight gain. Conversely, obesity might trigger alterations in the sympathetic regulation of cardiovascular function, thus favoring the development of cardiovascular complications and events. This article is aimed at reviewing the role of ANS in the pathophysiology of obesity, and thus to identify possible new therapeutic targets for the treatment of obesity and its complications.

## Role of the ANS in energy homeostasis

Body weight is regulated by a complex homeostatic system, whose main components are the modulation of appetite and satiety and the modulation of energy expenditure and energy storage in the adipose tissue. This homeostatic system is aimed at maintaining a stable body weight and requires the existence of a network of signals conveying information from the periphery to the central nervous system (CNS), where these signals are integrated and contribute to long-term and short-term regulation of body weight (Cummings and Schwartz, 2003). Peripheral signals involved in energy homeostasis can be classified as short-acting signals, such as gastric distension and gut hormone release, which are acutely affected by ingested nutrients and modulating satiety, and long-acting signals, such as leptin and insulin, which regulate overall body weight and adiposity.

**Figure 1:**
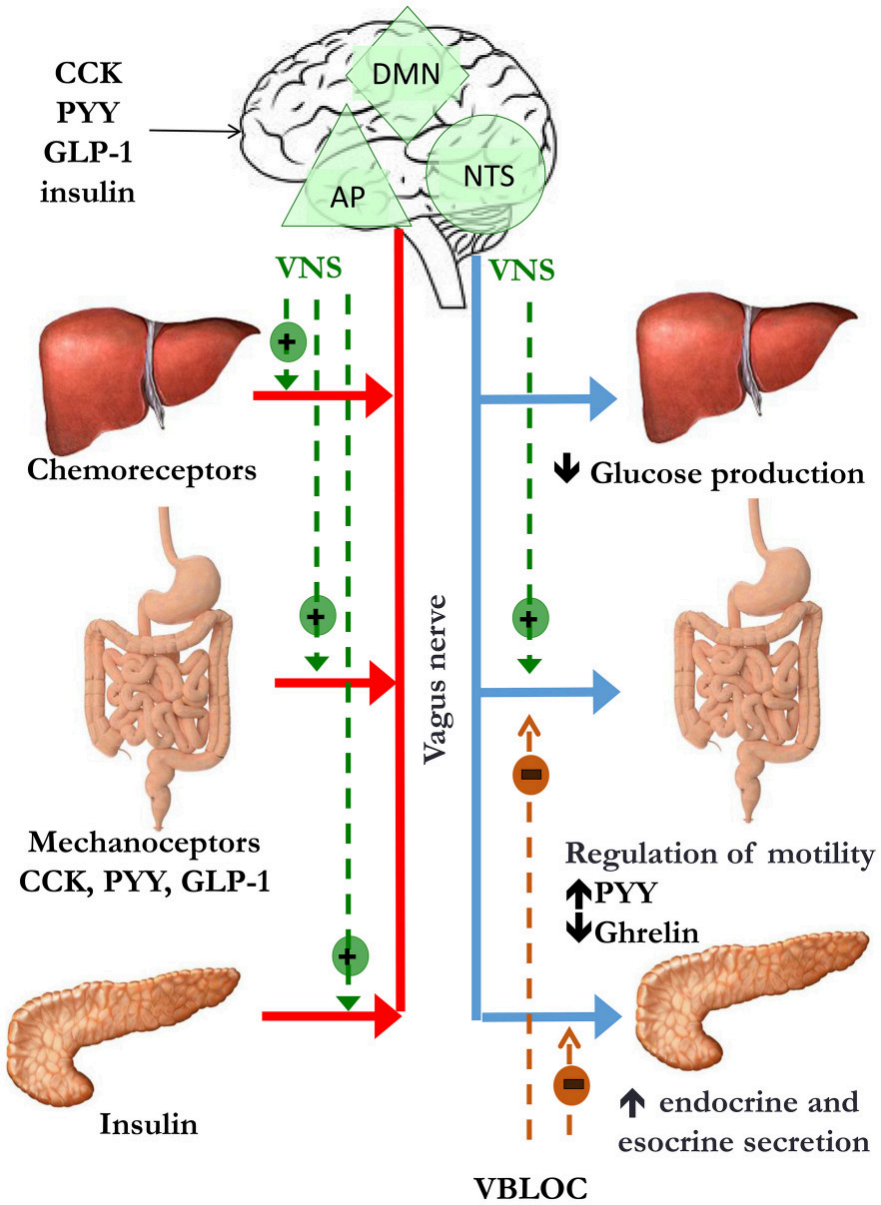
Peripheral signals of satiety and gastric emptying reach the nucleus of the solitary tract/area postrema complex (NST/AP) via afferent vagal nerves (red line). The NTS projects to the dorsal motor nucleus (DMN). This pathway modulates intestinal motility and secretion, glucose production and pancreatic secretion via efferent vagal nerves (blue line). The suggested site of action of vagal nerve stimulation (VNS) is indicated by the dotted green lines, while mechanism of weight loss hypothesized vagal nerve blockade includes decrease in gastric emptying, increase in gut hormones release and inhibition of pancreatic esocrine secretion (dotted orange lines).

It is clear that any dysfunction in the pathways involved in maintaining body weight homeostasis may lead to weight gain and obesity. The ANS plays a central role in the communication between the CNS and the gastrointestinal system either in short-term or in long-term regulation of body weight (Figure 1). Going into detail, vagal afferents to the brain are crucial for information transfer from gut hormones and CNS and as a mediator of sense of satiety after gastric distension.

### ANS and short-term regulation of body weight

The main mediators of short-term regulation of body weight through the sensation of satiety are:

- Gastric distension (mediated by vagal afferents) (Figure 1);
- Gut hormones release. Indeed the gastrointestinal tract, in addition to its primary role in digestion and adsorption of nutrients, regulates food ingestion by gut hormones. Interestingly, part of their action is mediated by vagal afferents. The action of gut hormones on vagal afferent neurons is now recognized to be an early step in controlling nutrient delivery to the intestine by regulating food intake and gastric emptying. Therefore, gut hormones and vagal afferent neurons have been considered playing an important role in the pathogenesis of obesity (Dockray, 2014).

Satiety is a result of neuro-humoral stimuli generated during food intake, leading to control of meal size and termination (Woods et al., 1998): thus it is not surprising that an altered sense of satiety has been involved in the pathogenesis of obesity. The main hypothalamic areas involved in the control of both hunger and satiety are the arcuate nucleus (ARC), the paraventricular nucleus, the dorsomedial and ventromedial hypothalamus, and the lateral hypothalamic area. These areas are influenced by different peripheral signals coming from the liver and gut, the endocrine pancreas and the adipocytes, which could act directly on neurons in the CNS or through afferent neurons. Indeed, the afferent vagal pathways are probably the most important link between the gut and the brain for satiety signal modulation (Berthoud, 2008a). Vagal afferent neurons receive post-ingestive information from the gastrointestinal tract by mechanoreceptor stimulation (Ikramuddin et al., 2014) in response to gastric distension, by gut hormone release in response to nutritional composition of food consumed, and by direct action of some nutrients, such as short chain fatty acids (Baskin et al., 1999; Obici et al., 2002; Brown et al., 2006; Capasso and Izzo, 2008; Shin et al., 2009; Scherer et al., 2011; Iwasaki et al., 2013). Finally, vagal afferents receive metabolic information by chemoreceptors located in the hepatoportal system (Yi et al., 2010; Figure 1). Signals from peripheral receptors reach via vagal afferents the nucleus of the solitary tract/area postrema (NTS/AP) complex in the brain stem, which integrates sensory information from the gastrointestinal tract and abdominal viscera and taste information from the oral cavity (Travers et al., 1987). NTS projects back to the gut via vago-vagal autonomic reflexes through the dorsal motor nucleus. The stimulation of this pathway leads to gut responses, including control of intestinal transit time and motility (i.e., delayed gastric emptying) (Forster et al., 1990), absorption rate and exposure of enteroendocrine cells (EECs) to nutrients, with changes in gastrointestinal hormones and pancreatic secretion, involved in satiety (Li and Owyang, 1994; Berthoud, 2008b).

### ANS and gut hormones

#### Cholecystokinin (CCK)

Cholecystokinin (CCK) is an anorectic hormone secreted by different tissues, including the I-cells of the small intestine (Buffa et al., 1976), with the main effect of reducing meal size and duration (Kissileff et al., 2003). Its release pattern suggests that CCK plays a role in meal termination and early phase satiety (Burton-Freeman et al., 2002).

CCK binds A-type receptors, found either in the periphery or in the brain, and B-type receptors, found only in the brain (Fink et al., 1998). CCK may act directly on the CNS (Blessing, 1997) and/or peripherally via vagal afferent fibers (Corp et al., 1993; Burdyga et al., 2003). Some authors reported that the main mechanism trough which CCK regulates food intake is the inhibition of gastric emptying (Moran and Kinzig, 2004). Furthermore, Wank (1995) and Granger et al. (1980) CCK induces gastrointestinal vasodilation acting on CCK-A receptors placed on abdominal vagal afferents projecting to NTS. This pathway involves also caudal and rostral ventrolateral medulla neurons, thus leading to suppression of sympathetic vascular tone (Sartor and Verberne, 2002, 2006, 2008). The role of alteration of CCK secretion in obesity is uncertain: indeed, obese patients exhibit higher CCK plasmatic levels that lean individuals, either in fasting conditions or after a high-fat meal (Little et al., 2005).

#### Peptide YY (PYY)

Peptide YY (PYY) is released by the L-cells of the gastrointestinal tract, in response to a meal in proportion to calories, and to luminal content of fatty acids, fibers and bile acid (Adrian et al., 1985; Onaga et al., 2002). Its actions in the brainstem and in the gut are mediated by Y_1_ and Y_2_ receptors (Yang, 2002). PYY acts mainly via the Y_2_ receptor (Dumont et al., 1995), identified on both intestinal vagal afferents and within the ARC: both pathways may thus be involved in the anorectic effects of Y_2_ receptor activation (Fetissov et al., 2004; Koda et al., 2005). Central and peripheral specific binding sites of PYY have been identified in NTS/AP and in dorsal motor nucleus (Parker and Herzog, 1999), as well as in in enterocytes, myenteric and submucosal neurons (Cox, 2007a,b). PYY release in the post-prandial period seems to be induced also by the indirect stimulation of endocrine L-cells through vagal neural pathways (Fu-Cheng et al., 1997; Lin and Taylor, 2004). In animal models, PYY release was blocked by atropine, a nicotinic ganglionic blocker (Lin and Taylor, 2004), while intravenous administration of bethanechol (a muscarinic cholinergic agonist) stimulated PYY release (Dumoulin et al., 1995). PYY acts also as a counterregulatory hormone for ghrelin release via growth hormone secretagogue receptor, expressed in the nodose ganglion of vagal nerves (Neary et al., 2003) and in the ARC. PYY plasma concentrations are lower in obese in comparison to lean individuals either in the fasting period (Batterham et al., 2003) or in the post-prandial period (le Roux et al., 2006). The latter phenomenon could be responsible of impaired satiety signal in obesity, since PYY infusion reduces caloric intake both in obese and lean individuals (Batterham et al., 2003). Experimental data suggest that electrical vagal stimulation may increase PYY secretion from the isolated ileum in pigs (Sheikh et al., 1989).

#### Pancreatic polypeptide (PP)

Pancreatic Polypeptide (PP) is secreted by cells located at the periphery of the pancreatic islets, in the esocrine pancreas and distal gut (Track, 1980; Ekblad and Sundler, 2002) in response to food intake. PP has inhibitory effects on gastric emptying, and delays the post-prandial rise in insulin (Schmidt et al., 2005). The vagal nerve controls both PP basal and post-prandial release. Surgical or pharmacological vagal blockade causes a marked reduction in meal-induced PP release in dogs (Niebel et al., 1987) and humans (Meguro et al., 1995).

The role of PP in obesity pathogenesis is controversial. Some authors reported a blunted post-prandial PP increase in obese individuals (Lassmann et al., 1980; Glaser et al., 1988), and no differences have been reported in circulating PP between obese subjects and lean individuals (Jorde and Burhol, 1984). However, since plasma PP concentrations are almost exclusively under vagal control, they can be used as an indicator of vagal activity in a number of experimental settings (Schwartz, 1983; Arosio et al., 2004).

#### Glucagon-like peptide-1 (GLP-1)

Glucagon-like peptide-1 (GLP-1) is an anorectic hormone, member of the incretin family. It is cleaved from preproglucagon within the intestine, where it is released by endocrine L-cells of the distal gut (Wettergren et al., 1997). GLP-1 levels rises post-prandially in response to a meal and fall in the fasting state. GLP-1 release is proportional to the calories ingested (Kreymann et al., 1987; Orskov et al., 1994) and it is particularly responsive to carbohydrates (Lavin et al., 1998) and fats (Frost et al., 2003). Some authors have suggested that circulating GLP-1 levels are reduced in obesity and normalized with weight loss (Verdich et al., 2001). GLP-1 mediates glucose-dependent insulinotropic effects in a number of species, including humans (Holst et al., 1987; Mojsov et al., 1987). Furthermore, it inhibits gastric acid secretion and gastric emptying (Imeryuz et al., 1997; Edvell and Lindstrom, 1999; Sheikh, 2013). The effects of GLP-1 on appetite regulation are mediated by the GLP-1 receptor. GLP-1 receptors are found not only in peripheral tissues (Bullock et al., 1996) but also in CNS areas (Kastin et al., 2002) involved in the regulation of satiety and induction of taste aversion, such as NTS/AP and ARC (Turton et al., 1996). In animal models GLP-1 actions on CNS seem to be mediated by afferent vagal fibers (Ronveaux et al., 2015). Indeed, vagotomy attenuates the satiating effect of GLP-1 (Nakabayashi et al., 1996; Abbott et al., 2005). Recent data showed that an intact vagal nerve is necessary for the inhibition of food intake by intravenous GLP-1 in human patients undergoing vagotomy and pyloroplasty (Plamboeck et al., 2013). Furthermore, some evidence suggest that GLP-1 crosses the blood brain barrier to act directly on CNS receptors (Kastin et al., 2002).

#### Ghrelin

Ghrelin is an orexigenic hormone, primarily secreted by endocrine cells in the oxyntic mucosa of the stomach. Ghrelin stimulates eating behavior and is involved in meal initiation; ghrelin suppression after a meal is crucial to provide a feedback signaling to brain and stop food intake (Kojima et al., 1999; Cummings et al., 2001; Tschop et al., 2001). Thus it is not surprising that obese individuals, though exhibiting lower fasting ghrelin levels than lean individuals, lack the physiological ghrelin suppression in the post-prandial phase: this phenomenon could lead to increased food consumption and, finally, obesity (English et al., 2002).

Ghrelin suppression after meals, which is crucial to reduce caloric intake, is induced by several factors include changes in plasma insulin, intestinal osmolarity, and enteric neural signaling, but a key role for vagal signaling has been also hypothesized (Date et al., 2002; Lee et al., 2002). Indeed in healthy humans vagal stimulation, achieved by modified sham feeding technique (in which nutrients are chewed and tasted but not swallowed) has an inhibitory effect on ghrelin release comparable to real feeding (Arosio et al., 2004; Heath et al., 2004).

Ghrelin plays also a role in long-term body weight regulation, acting as an adiposity signal, communicating the state of energy stores to the brain. Thus fasting ghrelin levels are reduced in obese individuals, and increase after weight loss (Cummings, 2006). However, gastric bypass is associated with markedly suppressed ghrelin levels: this phenomenon possibly favor a greater weight loss after this surgical procedure (Cummings et al., 2002).

#### Insulin

Insulin, beyond its established role in glucose (Obici et al., 2002) and lipid metabolism (Scherer et al., 2011), is also involved in satiety pathway acting on CNS. Chronic or acute intracerebroventricular administration of insulin reduces food intake and body weight in a variety of species. Insulin receptors are expressed in the CNS neurons, especially in the ARC (Plum et al., 2005), and participate in the food intake control (Baskin et al., 1999; Brown et al., 2006). On the other hand, insulin could act on its peripheral receptors located in the nodose ganglion (Iwasaki et al., 2013). Hyperphagia and obesity could be, at least in part, caused by impaired response to insulin of nodose ganglion neurons (Iwasaki et al., 2013).

Chronic hyperinsulinemia is a feature of obesity, aimed at restoring energy balance and limiting weight gain in a compensatory fashion. However, it may act as a maladaptive mechanism, inducing sympathetic overactivity (Landsberg, 1986).

#### Leptin

Leptin is a hormone released by the white adipose tissue (WAT), whose main actions are to suppress appetite and to regulate glucose metabolism (Elmquist et al., 1998; Elias et al., 2000). However, leptin pathways are involved also in energy expenditure control, as reviewed below. Leptin plasma levels decrease during fasting and increase after overfeeding, whereas leptin administration decreases food intake in animals and humans (Campfield et al., 1995; Heymsfield et al., 1999). The ARC is the most important site involved in leptin-related food intake (Satoh et al., 1997; Haynes, 2000). Within the ARC, two antagonistically acting neuronal populations, the neuropeptide Y (NPY) and proopiomelanocortinergic (POMC) neurons, were identified as immediate downstream targets of leptin. Even though leptin receptors are expressed on both neuronal populations, leptin stimulation of NPY neurons decreases their firing and attenuates food intake, whereas its actions on POMC neurons are opposite (Pandit et al., 2017).

While genetic syndromes characterized by leptin deficiency present hyperfagia and obesity (Zhang et al., 1994), most obese individuals rather have hyperleptinemia (Schwartz et al., 1997), due to desensitization of its own receptor (Considine et al., 1996).

SNS is involved in regulation of secretory function of WAT, especially for leptin secretion. Indeed, acute treatment with catecholamines in *in vitro* experimental human studies reduces circulating leptin through β1 and β2 receptors (Scriba et al., 2000). Furthermore, sympathetic activation induced by cold exposure induces not only increased metabolic rate and mobilization of free fatty acids, but also a rapid decrease in leptin gene expression and plasma leptin levels (Trayhurn et al., 1995).

### ANS and the long-term regulation of body weight

The ANS seems to play a role, though not entirely clear, in energy expenditure and storage. In humans, the energy is stored mainly in the WAT under the action of insulin, from where can be mobilized mainly by activation of SNS. Furthermore, SNS might increase energy expenditure by acting either on brown adipose tissue (BAT) thermogenesis or on the cardiovascular system: this neuronal pathway is modulated by leptin (Pandit et al., 2017)

#### The role of SNS in lipolysis

It is well known that lipolysis in the WAT is regulated by SNS and insulin, the principal initiator of lipolysis and a potent inhibitor of lipolysis respectively (Goodridge and Ball, 1965; Prigge and Grande, 1971). Indeed, sympathetic nerve stimulation results in fatty acid release (Rosell, 1966), while sympathetic or ganglionic blockade inhibits lipid mobilization (Gilgen et al., 1962). On the other hand, adrenal medullary catecholamines have no effects on lipid mobilization (Takahashi and Shimazu, 1981), confirming that lipolysis is induced by increased SNS outflow directed to WAT (Rebuffe-Scrive, 1991). Kreier et al. (2002) hypothesized also a parasympathetic innervation of WAT in animal models, possibly modulating insulin-mediated glucose uptake and free fatty acid metabolism in an anabolic way, thus promoting lipid accumulation. According to this hypothesis, lipid accumulation in obesity could be due either to a decrease in SNS activity or by an increase in parasympathetic activity (Bartness, 2002). However, other studies failed to demostrate parasympathetic innervation in WAT (Giordano et al., 2006).

#### The role of SNS in energy expenditure

Total energy expenditure is composed of resting metabolic rate (including cardiorespiratory work and the maintenance of transmembrane ion gradients at rest), physical activity and thermogenesis (shivering and non-shivering), and the termic effect of food. SNS activation induces total energy expenditure, either increasing cardiorespiratory work or increasing thermogenesis.

It is well known that the SNS plays a pivotal role in both blood pressure and metabolic homeostatic control by regulating cardiac output, peripheral vascular resistance, and heat production, which account for a large fraction of resting metabolic rate (Goran, 2000). Indeed, pharmacological adrenergic blockade is able to reduce resting energy expenditure (Welle et al., 1991; Monroe et al., 2001; Shibao et al., 2007).

At variance to what was previously thought, BAT is not present only in children, but also in lean and obese adult humans (Virtanen et al., 2009). Its main function is to increase energy expenditure by inducing cold- or diet-stimulated heat production (van der Lans et al., 2013), and by uncoupling oxidative phosphorylation from ATP synthesis through the uncoupling protein-1 in BAT mitochondria (Cannon and Nedergaard, 2004; Saito, 2013). Functional BAT in adults is detectable after exposure to mild cold (Saito et al., 2009) and its activity is inversely related to body mass index and body fat percentage (van Marken Lichtenbelt et al., 2009). Lean subjects increase energy expenditure in response to mild cold, whereas obese subjects have a blunted cold-induced thermogenesis (Wijers et al., 2010).

**Figure 2:**
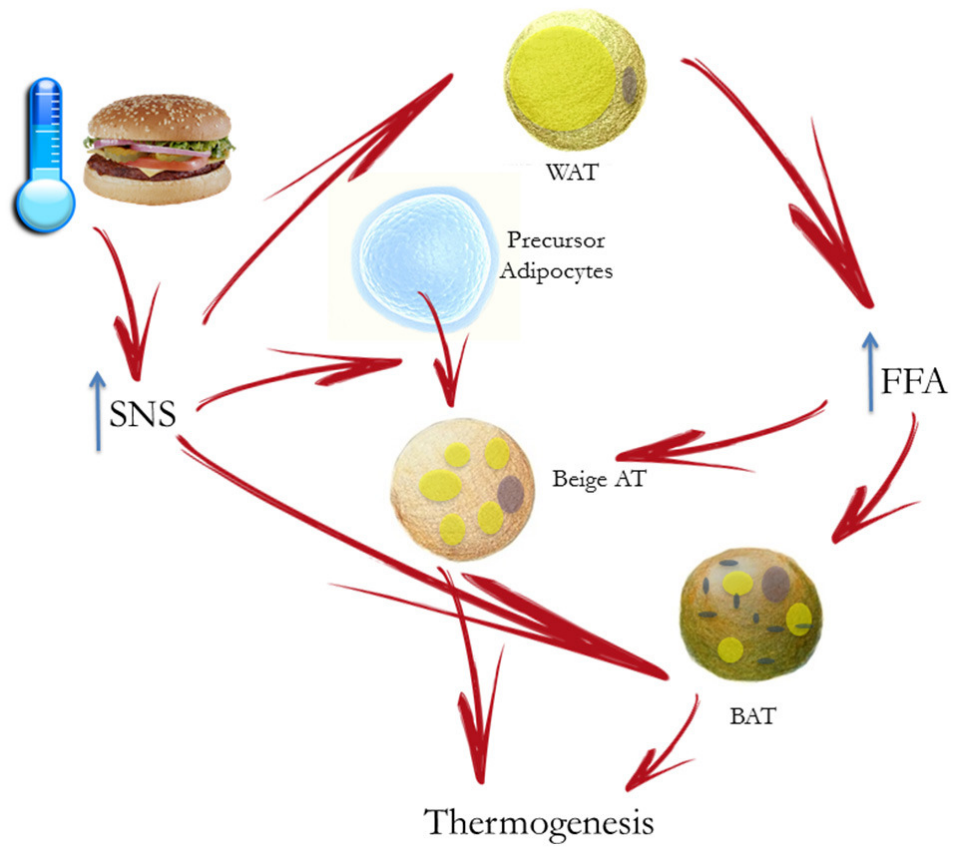
Cold- or diet-stimulated sympathetic activation results in mobilization of free fatty acids (FFA) by white adipose tissue (WAT) and regulation of brown adipose tissue (BAT) thermogenesis. The principal substrate for BAT is constituted by fatty acids to increase energy expenditure inducing heat production. Chronic sympathetic nervous system (SNS) activation also induces the conversion of “beige” adipose tissue in WAT, which also contribute to adaptive thermogenesis.

BAT thermogenesis is regulated by sympathetic nerves. As previously stated, sympathetic activation results in mobilization from WAT of fatty acids, which are then used by BAT to dissipate energy as heat (Figure 2). As far as sympathetic control is concerned, patients with surgical unilateral sympathectomy show a detectable uptake of 18F-*f* luorodeoxyglucose (*1*8F-FDG) in BAT by positron emission tomography on the unaffected side, but not on the side of surgical sympathectomy (Lebron et al., 2010). Administration of β-adrenergic receptor blockade reduces BAT 18F-FDG uptake (Soderlund et al., 2007) in patients with known or suspected cancer as well as in a patient with paraganglioma, a condition characterized by a massively increased metabolic BAT activity, induced by excess circulating catecholamines (Cheng et al., 2012). The role of α-receptors and α-blockade is less clear. In a patient with catecholamine-secreting paraganglioma, BAT 18F-FDG uptake was suppressed after α-blockade (Sondergaard et al., 2015). The sympathomimetic drug ephedrine activates BAT in lean but not in obese subjects, though the degree of activation is substantially lower than observed after cold exposure (Carey et al., 2013). Conversely, the effect of parasympathetic nervous system on BAT appears to be indirect. Indeed, in animal models, the suppression of NE release in BAT, induced by ghrelin infusion, is abolished after vagotomy (Mano-Otagiri et al., 2009). The authors hypothesized that the vagal nerve mediates the peripheral action of ghrelin, thus inhibiting sympathetic traffic directed to BAT. The interaction between vagal and BAT activity was confirmed in patients undergoing vagal nerve stimulation (VNS) for refractory epilepsy: VNS induced a BAT-mediated increase in energy expenditure (Vijgen et al., 2013).

Furthermore, chronic sympathetic activation produces a remarkable induction of uncoupling protein1-positive brown-like adipocytes in white fat pads, called “beige” adipose tissue, which also contribute to adaptive thermogenesis and body fat reduction (Cousin et al., 1992; Inokuma et al., 2006; Figure 2). In humans it has been suggested that BAT is mostly composed of beige cells and is inducible in response to appropriate sympathetic stimulation. In healthy human participants, with undetectable or low BAT activity, daily 2-h cold exposure at 17°C for 6 weeks resulted in increased BAT activity. Changes in BAT activity and body fat content were negatively correlated (Yoneshiro et al., 2013).

It is important to note that leptin has a crucial role in regulation of energy expenditure through SNS. Indeed, leptin has been shown to increase energy expenditure acting both on the cardiovascular system and BAT thermogenesis via the hypothalamus (Pandit et al., 2017). The ARC represents the main site of action of leptin on SNS. In particular, CNS leptin administration does not affect sympathetic nerve activity after ARC destruction (Haynes, 2000). However, Fischer showed that leptin may increase energy expenditure by inducing a pyrexic increase in body temperature by reducing heat loss, rather than affecting BAT thermogenesis (Fischer et al., 2016).

On the other hand, in animal studies leptin administration in different CNS areas increases sympathetic outflow to the kidneys, the adipose tissue, the skeletal muscle vasculature and adrenal glands (Dunbar et al., 1997; Elmquist et al., 1997; Haynes et al., 1997), thus causing an increase in energy expenditure (Woods and Stock, 1996) and in sympathetic vasomotor activity (Marsh et al., 2003). The latter mechanism is involved in pathogenesis of obesity –induced hypertension, as explained later (see Section Sympathetic Overactivity in Obesity).

Taken together, these results suggest that BAT thermogenesis is an appealing target in obesity treatment. However, while promising evidence in experimental animals demonstrate that it is possible to impair BAT thermogenesis (i.e., by beta-adrenergic blockade), no intervention has so far been able to increase it (Tupone et al., 2014).

## Sympathetic overactivity in obesity

An increased SNS activity has been demonstrated in obese patients, particularly in the muscle vasculature and in the kidneys, possibily contributing to increased cardiovascular risk. Though SNS activation is similar in hypertensive and normotensive obese individuals, sympathetic contribution to blood pressure via vasoconstriction is greater in the hypertensive ones, confirming a role for sympathetic activation in the pathogenesis of obesity-related hypertension. Conversely, sympathetic overactivity is not effective in favoring energy expenditure and thus weight loss. Selective leptin resistance, obstructive sleep apnea syndrome, hyperinsulinemia and low ghrelin levels are possible mechanisms underlying sympathetic activation in obesity. Weight loss is able to reverse metabolic and SNS alterations associated with obesity.

### Patterns of SNS activation in obesity

**Figure 3:**
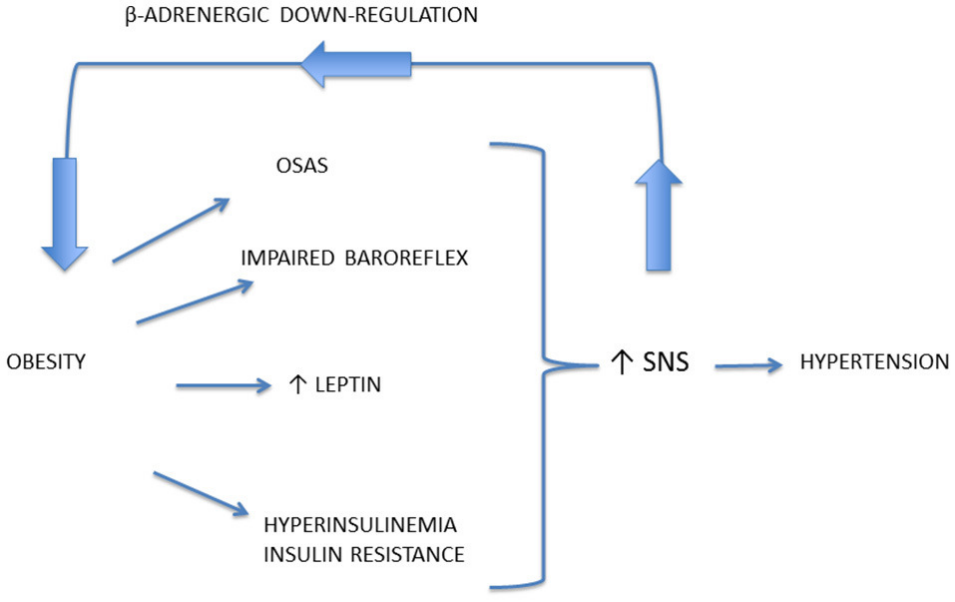
Mechanisms responsible for the occurrence of sympathetic activation in obesity-related hypertension. Prolonged sympathetic nervous system (SNS) overactivity might induce weight gain, due to downregulation of beta-adrenoceptors, thus reducing the capacity to dissipate excessive calories.

It is well known that excess weight is associated with ANS dysfunction, and particularly with increased sympathetic traffic. Landsberg was the first researcher speculating that increased SNS activity in response to weight gain is an adaptive mechanism to increase resting energy expenditure and promote restoration of the antecedent weight (Landsberg, 1986, 2001), while other authors suggested that prolonged sympathetic overactivity might induce weight gain, due to reduced capacity to dissipate excessive calories, mediated by downregulation of β adrenoceptors (van Baak, 2001; Feldstein and Julius, 2009; Figure 3). On the other hand, some authors suggested that a reduced sympathetic activity is rather implied in obesity pathogenesis, inducing a lower rate of thermogenesis and a positive energy balance (Bray, 1991). However, several studies conducted with sophisticated techniques supported the Landsberg's hypothesis of SNS overactivity in obese individuals, with or without hypertension (Landsberg, 1986).

It is important to underline that obesity causes a selective and differentiated increase in sympathetic activity rather than generalized SNS activation. This crucial issue has been investigated by techniques such as microneurography, which allows recording directly spontaneous efferent activity of post-ganglionic SNS fibers controlling muscle vascular tone (Vallbo et al., 2004), and regional NE spillover, which is crucial in order to investigate organs like heart and kidney, whose efferent nerve traffic is not directly recordable in humans. Several studies highlighted that obesity is characterized by SNS overactivity directed to the muscle vasculature by means of microneurography (Grassi et al., 1995, 2004; Alvarez et al., 2004). In obese individuals, increased MSNA is obtained by recruitment of additional nervous fibers, as demonstrated by single fiber recordings, at variance to the increased firing frequency observed in essential hypertension (Lambert et al., 2007). MSNA values, although increased in both central and peripheral obesity, are greater in individuals with an abdominal or central distribution of body fat (Grassi et al., 2004), particularly with visceral obesity (Alvarez et al., 2004). Several reflex abnormalities were shown in obesity, such as impaired baroreflex sensitivity (Grassi et al., 1995), central chemoreflex hypersensitivity (Narkiewicz et al., 1999a) and blunted muscle metaboreflex (Negrao et al., 2001); conversely, MSNA responses to mental stress and cold pressure test were similar in obese and in lean subjects (Kuniyoshi et al., 2003).

Furthermore, an increased adrenergic tone in the renal district was also demonstrated, while the sympathetic outflow to the heart is not elevated or even reduced, as demonstrated by cardiac norepinephrine spillover (Esler et al., 2006). It has been hypothesized that cardiac sympathetic tone is reduced in human obesity in response to volume overload (Messerli et al., 1983), in part induced also by sodium retention mediated by high renal SNS activity (DiBona, 1992). An altered autonomic modulation of heart rate has been also demonstrated by the technique of spectral analysis of heart rate variability (Hirsch et al., 1991; Tonhajzerova et al., 2008), with conflicting findings (Matsumoto et al., 1999; Antelmi et al., 2004).

An impaired autonomic regulation in the post-prandial phase has also been suggested. As mentioned above, SNS inhibition is the physiological response to fasting, in order to limit weight loss during starvation (Young and Landsberg, 1977), while food ingestion, particularly of carbohydrate-rich food, induces an increase in SNS activity (Young and Landsberg, 1977; Welle, 1995). This physiological response is blunted in obese individuals in comparison to lean individuals, though energy expenditure was similar and no correlation between SNS activity and the thermic effect of the food has been demonstrated (Tentolouris et al., 2003). The blunted post-prandial increase in sympathetic tone, demonstrated also in adult obese individuals (Xu et al., 2014) may thus represent a mechanism of inhibition of post-prandial thermogenesis, thus favoring weight gain, though conflicting data exist (Emdin et al., 2001). However, these results do not allow drawing firm conclusions, since only autonomic modulation of heart rate has been explored, which may not represent sympathetic traffic directed to the adipose tissue.

Finally, it is important to note that sympathetic overactivity characterizing obesity has deleterious cardiovascular consequences, including the development of hypertension, but it is not effective in increasing energy expenditure and favoring weight loss as expected (see Section Role the ANS in Energy Homeostasis). Indeed, acute ganglionic blockade (Shibao et al., 2007), did not change energy expenditure in individuals with central obesity, supporting the Landsberg's hypothesis of sympathetic activation in obesity as a compensatory but ineffective strategy induced by weight gain. However, preliminary data suggest that contribution of SNS after gastric bypass might be very small: this fact might make more difficult to maintain weight loss after surgery (Curry et al., 2013).

### Mechanisms of sympathetic activation in obesity and obesity-related hypertension

Adrenergic activation plays an important role in pathophysiological mechanisms underlying the development, maintenance, and progression of essential hypertension (Grassi et al., 2015) and is suspected to contribute in particular to the development of hypertension in obese humans (Hall et al., 2012). Julius et al. first proposed that increased sympathetic activity in hypertension was the primary defect leading to insulin resistance and weight gain in obese adults (Julius et al., 2000). In young overweight individuals, SNS activity is directly related to the degree of cardiac, renal, and vascular dysfunction, suggesting that sympathetic neural drive may be a major player in CV risk development (Lambert et al., 2010).

Mechanisms underlying obesity-related hypertension are not fully understood. Indeed, a great importance has been given to activation of renal sympathetic nerves, causing sodium retention, increased renin secretion, and impaired renal-pressure natriuresis (Hall et al., 2012). Though renal NE spillover is similar in normotensive and hypertensive obese individuals, an exaggerated effect of SNS activation has been reported. Indeed Shibao and coauthors demonstrated that after ganglionic blockade with trimethaphan, hypertensive obese patients exhibited a greater BP fall than the normotensive ones (Shibao et al., 2007). Central mechanisms may be relevant in obesity-related hypertension and include activation of leptin and POMC pathway, and obstructive sleep apnea syndrome, with activation of chemoreceptor-mediated reflexes related to intermittent hypoxia (Figure 3). Furthermore, among peripheral mechanisms of sympathetic activation, hyperinsulinemia might play a role.

#### Leptin

As already mentioned, leptin has central sympathoexcitatory effects, demonstrated in a number of experimental studies (Haynes et al., 1999; Lim et al., 2013). Indeed, obese mice with leptin or leptin-receptor deficiency showed no increase in arterial pressure (Mark et al., 1999). The sympathoexcitatory and hypertensive effect of leptin seems to be mediated by melanocortin-4 receptor (MC4R) (Tallam et al., 2005). These findings were confirmed also in MC4R deficient humans, who show a low prevalence of hypertension, despite the presence of severe obesity (Greenfield et al., 2009).

Based on this piece of evidence, Mark et al. suggested that some forms of obesity may be characterized by a “selective leptin resistance,” limited to its favorable metabolic effects (satiety and weight loss), while its sympathoexcitatory effects on the cardiovascular system are maintained (Correia et al., 2002; Mark et al., 2002; Rahmouni et al., 2005). In humans, a number of studies confirmed the association between leptin and hypertension. Human leptin deficiency was associated with early-onset morbid obesity and metabolic syndrome without SNS activation or hypertension (Ozata et al., 1999). Conversely, higher leptin levels in obese hypertensive in comparison to obese normotensive individuals have been reported (Kunz et al., 2000; Golan et al., 2002). Furthermore, in the Copenhagen City Heart Study increased plasma leptin levels predicted the risk of developing hypertension (Asferg et al., 2010). However, acute of chronic administration of leptin in humans failed to induce a sustained BP or SNS activity increase, thus the role of leptin in causing sympathetic activation in obesity still need to be fully clarified (Mark, 2013).

#### Obstructive sleep apnea syndrome (OSAS)

OSAS is a condition characterized by repetitive episodes of upper airway narrowing or occlusion, causing chronic intermittent hypoxia and sleep fragmentation (Dempsey et al., 2010). Obesity is a major risk factor for OSAS, which in turn may induce BP increase not only during nighttime but also during daytime (Brooks et al., 1997). The role of OSAS as a determinant of sympathetic overactivity has been reported not only in obese (Somers et al., 1995; Narkiewicz et al., 1998) but also in lean subjects (Grassi et al., 2005). Interestingly, some authors suggest that obesity *per se* is not associated to increased sympathetic traffic to the muscle vasculature, but this alteration is present only when obesity is accompanied by OSAS (Narkiewicz et al., 1998). Mechanisms of hypertension development during OSAS include sympathetic activation due to chemoreflex activation, secondary to repetitive hypoxic episodes at nighttime, but also alterations in vascular function and structure caused by oxidative stress and inflammation (Bruno et al., 2013). A sustained reduction in MSNA was demonstrated in normotensive patients with OSAS after both 6 and 12 months of continuous positive airway pressure therapy (Narkiewicz et al., 1999b).

#### Insulin

Some authors suggest that chronic hyperinsulinemia may act as a maladaptive mechanism, inducing SNS overactivity in obesity (Landsberg, 1986, 2001). However, this hypothesis has not been supported by later studies. Indeed, insulin administration has a direct vasodilatory effect during acute euglycemic hyperinsulinemic clamp: thus the increase in MSNA and norepinephrine levels reported in healthy individuals and hypertensive patients may be a consequence of baroreflex activation (Rowe et al., 1981; Anderson et al., 1991, 1992). However, a modest increase in BP was observed in healthy individuals when supraphysiological insulin concentrations are obtained (Rowe et al., 1981). Interestingly, in elderly subjects with normal BP, acute elevations of plasma insulin during hyperinsulinemic/euglycemic clamp caused vasoconstriction, accompanied by a blunted increase in norepinephrine and heart rate, as compared to young individuals, while no changes in BP were observed in either group. The authors suggested that the insulin-induced vasoconstriction is not due to exaggerated insulin-induced sympathetic activation but rather to a reduction in the vasodilator action of insulin (Hausberg et al., 1997). Despite hyperinsulinemia, intracerebroventricular administration of insulin antagonists did not affect renal sympathetic nerve activity in experimental animals, adding to the evidence that insulin does not promote obesity hypertension by chronically stimulating the SNS (Lim et al., 2013).

#### Ghrelin

Beyond its established role in appetite regulation, ghrelin has beneficial effects on blood pressure (BP) and cardiovascular function (Virdis et al., 2016), possibly modulating ANS activity. In experimental animals, intracerebral infusion of ghrelin reduced BP; however, it is still not clear whether this effect was mediated by modulation of sympathetic traffic (Matsumura et al., 2002; Prior et al., 2014). Lambert et al. investigated the effects of supraphysiological doses of intravenous ghrelin in lean and obese individuals. Ghrelin did not influence SNS activity controlling resting calf vascular tone; however, ghrelin infusion blunted BP and muscle sympathetic nerve activity (MSNA) responses to acute mental stress after short-term ghrelin infusion either in lean or obese individuals (Lambert et al., 2011).

### Effect of weight loss on the SNS

Several studies have shown that sympathetic activation reported in obese subjects is reversed by weight loss (Muscelli et al., 1998; Nault et al., 2007; Perugini et al., 2010). This topic is extensively reviewed elsewhere (Lambert et al., 2015). Straznicky reported a marked sympathoinhibition secondary to diet-induced weight loss, evaluated by MSNA and whole-body plasma norepinephrine spillover rate (Straznicky et al., 2005). However, bariatric surgery is the most effective treatment for obesity, allowing to achieve up to 70% of excess weight loss (Buchwald et al., 2004). It is also well known that bariatric surgery improves the main defects responsible for obesity-associated hyperglycaemia, namely insulin resistance and beta-cell dysfunction (Ferrannini, 1998; Nannipieri et al., 2011). Few data explored the role of bariatric surgery in reduction of SNS activity. Pontiroli et al. showed a restoration of sympathovagal balance evaluated by heart rate variability in 24 subjects with severe obesity 6 months after gastric banding (Pontiroli et al., 2013), while Lips et al. showed an improvement in heart rate variability, although explored only in the time domain, after 3 months very low-calorie diet or gastric bypass (Lips et al., 2013). However, these two studies, using spectral analysis of RR interval, did not provide a measure of sympathetic activity. In 23 severely obese, non-diabetic, individuals, MSNA was measured before and after 10% weight loss induced by laparoscopic adjustable gastric band. Noteworthy, a significant reduction in BP, MSNA, fasting insulin and creatinine clearance was found, whereas cardiac and sympathetic baroreflex sensitivity were improved (Lambert et al., 2014). Seravalle et al. evaluated the effect of weight loss secondary to sleeve gastrectomy or caloric-restricted diet on the ANS. Six months after surgery, waist circumference, leptin levels and MSNA were reduced in the surgery group, which persisted 12 months after surgery (Seravalle et al., 2014). Conversely, insulin sensitivity, evaluated by Homeostatic Model Assessment (HOMA) index, was reduced after 6 months, but returned to pre-surgery values after 12 months, suggesting that sympathetic deactivation induced by weight loss might not influence insulin sensitivity (Seravalle et al., 2014). However, this conclusion is limited by the fact that HOMA index is a rough index of insulin sensitivity; furthermore, since it is derived from fasting insulin and glucose levels, it is related to hepatic insulin sensitivity rather than peripheral insulin sensitivity, which is conceivably more influenced by changes in sympathetic tone.

SNS activity after gastric bypass surgery seem to be lower than those of obese individuals and thus might blunt energy expenditure, with negative consequences for weight maintenance (Curry et al., 2013). We do not know whether different interventions, i.e., sleeve gastrectomy might lead to the same phenomenon.

Finally, it is important to note that the surgical procedure *per se* might have a direct impact on the autonomic innervation of the gastrointestinal tract. During surgery, sleeve gastrectomy and Roux-en-Y *gastric bypass* (RYGB) may damage the gastric branches of the vagal nerve in a different manner. Infact in the sleeve gastrectomy the stomach is cut longitudinally, damaging the very distal branches of the gastric vagal nerve, while in the RYGB the stomach is cut transversely, resulting in a damage of the gastric vagal branches very close to their origin from the esophageal plexus (Ballsmider et al., 2015). Thus, it is conceivable that the effects of bariatric surgery on brain-gut axys may be influenced by the surgically-induced anatomical alterations, which may affect the integrity of vagal innervation between the hindbrain feeding centers and the gastrointestinal tract.

## The ANS as a therapeutic target in obesity

Based on the physiopatological background above described, it is clear the modulation of ANS may induce weight loss and/or reduce cardiovascular risk in obese patients. VNS, achieved by implantable or transcutaneous devices, has been associated with a significant weight loss in small, non-randomized pilot studies. Vagal nerve blockade yelded either neutral or positive effects in term of weight loss in small sham-controlled studies, but even in this case further evidence is needed. Sympathetic inhibition accompanied weight loss achieved by diet or surgery. Interventions targeting SNS are able to improve cardiometabolic profile in obese individuals.

### Vagal modulation

Since vagal afferents convey to the CNS the gastric distension signal and satiety signals evoked by gut hormones, it is not surprising that vagal stimulation has been proposed as a weight loss intervention. Several studies, carried out in obese animals, showed that VNS suppressed food intake and weight gain. Bugajski et al. suggested that VNS, achieved by implantable electronic devices, mimics activation of gastric mechanoceptors and jejunal chemoceptors, thus resulting in decreased food intake and weight loss in obese rats (Bugajski et al., 2007; Figure 1). The limitations of this study are the monolateral VNS and the use of constant voltage stimulation (Bugajski et al., 2007). Bilateral VNS with constant current stimulation induced stable weight loss in obese minipigs (Val-Laillet et al., 2010). Furthermore, patients treated with vagal stimulation for severe depression experienced a relevant weight loss (Pardo et al., 2007) (Table 1). However, this approach is limited by its high cost and invasiveness, potential need for reintervention for mechanical failure and/or battery re-placement, and side effects (Ventureyra, 2000). More recently, transcutaneous auricular VNS (taVNS) has been proposed to treat disorders such as epilepsy (Miro et al., 2015) and depression, drawing inspiration from auricular acupuncture of traditional chinese medicine (Rong et al., 2016). The rationale for using taVNS is that anatomical studies showed that the ear is the only place on the surface of the human body where afferent vagal nerve distribution is present (Wang et al., 2014). Indeed, a branch of the vagal nerve provides sensory innervation of the “cymba conchae” of the external ear (Peuker and Filler, 2002). Thus, the direct stimulation of the afferent vagal nerve fibers on the ear may produce similar effects as classic VNS without the burden of surgical intervention (Henry, 2002). Indeed, cymba conchae stimulation of auricolar vagal branch activated the NTS and other vagal projections within the brainstem and forebrain in healthy adults (Frangos et al., 2015). Furthermore, in a pilot randomized clinical trial, Huang et al reported an improvement of in the 2-h glucose tolerance and systolic BP in after a 12-week treatment with taVNS in comparison with sham technique (Huang et al., 2014) (Table 1). Finally, taVNS is able to acutely reduce MSNA and shift cardiac autonomic function toward parasympathetic predominance in healthy volunteers (Clancy et al., 2014). These promising findings suggest that in obese and glucose-intolerant individuals, taVNS may not only restore insulin resistance and secretion, but also counteract obesity-related autonomic dysfunction (Lambert et al., 2010; Seravalle et al., 2014) and thus play a role in reducing its cardiovascular burden.

**Table 1:** Human studies investigating the role of VNS in weight loss and glucose control.

**Study**	**Population**	**VNS duration**	**Clinical endpoint**	**Results**
Pardo et al., 2007	14 patients with resistant depression	6–12 months	Change in level of depression and weight loss	Mean weight loss—7 kg; BMI change—2 kg/m^2^
Huang et al., 2014	70 IGT subjects randomly assigned to the taVNS group or sham taVNS group 30 IGT controls without device	6–12 weeks	2-h plasma glucose levels (2hPG) OGTT at 6 weeks and 12 weeks.	Reduction in 2 hPG in taVNS vs sham taVNS *p* = 0.004

On the other hand, gastric emptying is under the control of vagal efferent fibers. Vagotomy, in experimental animals (Smith et al., 1983) as well as in humans (Kral, 1978) is able to delay gastric emptying and impair gastric accommodation to food, thus inducing weight loss. Since pancreatic secretion is under vagal control, interruption of vagal efferent fibers induces malabsorption (Camilleri et al., 2008). Furthermore, vagotomy in rats prevents the physiological ghrelin increase in fasting conditions (Williams et al., 2003). Thus, intermittent electric stimulation of vagal fibers, inducing blockade of the neural transmission, has been tested as a novel weight-loss intervention (Table 2).

**Table 2:** Human studies investigating the role of vagal nerve blockade (VBLOC) in weight loss, glucose control and caloric intake.

**Study**	**Population**	**VBLOC duration**	**Clinical endpoint**	**Results**
Camilleri et al., 2008	31 obese subjects	6 months	% excess weight loss (%EWL) and caloric intake	EWL 14.2% vs. baseline (*p* < 0.001) Caloric intake decreased by 30% (*p* < 0.01)
EMPOWER study Sarr et al., 2012	192 obese subjects with VBLOC 102 obese subjects with device with a lower charge delivery	12 months	% excess weight loss (%EWL)	EWL 17 ± 2% in VBLOC vs. 16 ± 2% in device with a lower charge delivery (*p* = ns)
Shikora et al., 2013	26 obese subjects with type 2 diabetes with VBLOC	12 months	% excess weight loss (%EWL) and glucose control	EWL 25 ± 4% (*p* < 0.0001) and mean HbA1c reduction −1 ± 0.2% (*p* < 0.02) vs. baseline
ReCharge study Ikramuddin et al., 2014	162 morbid obese subjects with VBLOC 77 morbid obese subjects with sham device	12 months	% excess weight loss (%EWL)	EWL 24,4% in VBLOC vs. 15,9% in sham device (*p* = 0.002)

The EMPOWER study evaluated the effects of intermittent, bilateral blockade of bilateral subdiaphragmatic vagal nerves to stop both ascending and descending neural traffic, speculating its involvement in satiety, reduced food intake and weight loss in morbid obese individuals (Figure 1). However, despite the solid scientific background linking vagal activity and obesity, extensively described in the previous sections, the EMPOWER study yelded negative results: vagal blockade induced a similar weight loss than the control group, which had the same device with a lower charge delivery; interestingly, weight loss was related to device use time in both groups, suggesting that what was supposed to be a sham therapy was active as well (Sarr et al., 2012). This hypothesis is confirmed by the ReCharge study, in which vagal nerve blockade was obtained by using a device that delivered at least 12 h of therapy per day and was compared a sham control device that had no possibility of delivering therapy. Individuals undergoing vagal blockade therapy achieved a greater weight loss than the sham control group, although the pre-established efficacy outcomes were not achieved (Ikramuddin et al., 2014) (Table 2).

### Sympathetic modulation

Given the above-described role of SNS in the pathophysiology of obesity and its cardiovascular consequences, SNS inhibition is considered a potential therapeutic target in obesity. As reviewed above, it is important to underline that interventions aimed at inducing weight loss by diet or surgery are able to achieve a significant reduction in SNS tone, in particular in the muscle vasculature (Lambert et al., 2015).

Indeed, a number of mechanistic studies demonstrated that acute pharmacologic ganglionic blockade by trimetaphan is able to reduce blood pressure (Shibao et al., 2007), to improve insulin sensitivity (Gamboa et al., 2014) and to reverse endothelial function (Gamboa et al., 2016) in obesity, in particular if associated with hypertension. However, ganglionic blockers cannot be used chronically, given their unfavorable profile in terms of adverse effects.

A significant antihypertensive effect of a combined α and ß-blockade has been reported in dietary mediated obesity in dogs consuming high fat diets (Hall et al., 2001) and in obese individuals in which a greater reduction in BP in comparison to lean subjects was reported after 1 month of treatment (Wofford et al., 2001). Adrenergic blockade produced a significantly greater decrease in BP in obese than in lean patients with hypertension (Wofford et al., 2001), in line to the results reported with ganglionic blockade (Shibao et al., 2007). A study suggested also that the use of a BP-lowering central sympatholytic drug, moxonidine, might induce a small but significant weight loss, together with a reduction in blood pressure, triglycerides and fasting blood glucose (Chazova and Schlaich, 2013), though another study failed to demonstrate any impact on insulin sensitivity (Masajtis-Zagajewska et al., 2010). In contrast, β-blockers may exert negative or neutral effects on body weight and lipid and glucose profile (Lambert et al., 2015). However, some authors suggest that β-blockers may be first-choice drug in the treatment of hypertension in young adults, which is mainly linked to sympathetic overactivity due to overweight and obesity (Cruickshank, 2017).

In the past decade, great interest has been placed in device-based therapies targeting SNS for the treatment of refractory hypertension, such as renal denervation and baroceptor activating therapy (Bruno et al., 2013). Given the presence of sympathetic activation in obesity and its possible role in pathogenesis of obesity-associated hypertension, as described above, it may be expected that sympathetic inhibition might have a relevant impact in obese patients. Indeed, renal denervation seems able to restore insulin sensitivity in obese dogs (Iyer et al., 2016) but not in obese hypertensive mice (Asirvatham-Jeyaraj et al., 2016). Bilateral renal denervation greatly attenuated sodium retention and hypertension in obese dogs fed a high-fat diet (Kassab et al., 1995).

Glucose tolerance and glycemic control was significantly improved 3 and 6 months after renal denervation in 10 patients with resistant hypertension and OSAS: in this study, BP, but not BMI, was significantly reduced (Witkowski et al., 2011). This finding was confirmed in a larger cohort of resistant hypertensive patients, in whom renal denervation induced a reduction in blood fasting glucose, insulin, and HOMA-IR after 3 months (Mahfoud et al., 2011). However, the BP-lowering effect of such procedures has been recently questioned; furthermore, obese patients seem to benefit less of renal denervation in terms of BP reduction (Id et al., 2016).

## Conclusions

In conclusion, obesity is accompanied by increased morbidity and mortality, mostly related to cardiovascular disease, and represents a major issue for global healthcare. Thus, the study of mechanisms underlying its pathogenesis is crucial to identify novel targets for its treatment. The ANS plays a major role in the integrated short-term regulation of weight, modulating the satiety signal and energy expenditure. The afferent vagal pathways are probably the most important link between the gut and the brain and interact in a complex way with gut hormones. SNS has the physiological function of increasing lipolysis and energy expenditure, through sympathetic innervation in white and brown adipose tissue; thus it is abnormally activated in obesity in a compensatory but ineffective fashion. Sympathetic activation may favor the development of hypertension and organ damage in obesity and lead to overt cardiovascular disease. Though preliminary clinical trials exploring autonomic modulation as a treatment for obesity yelded contrasting results, mechanistic and physiopathological studies strongly support this therapeutic strategy as an appealing and promising approach for obesity treatment.

## Author contributions

DG drafted the manuscript. RMB designed and reviewed critically the article. MN, GI, and ST reviewed critically the manuscript.

### Conflict of interest statement

The authors declare that the research was conducted in the absence of any commercial or financial relationships that could be construed as a potential conflict of interest.
